# Analysis of the implementation of an innovative IT solution to improve waiting times, communication with primary care and efficiency in Rheumatology

**DOI:** 10.1186/s12913-021-07455-4

**Published:** 2022-01-12

**Authors:** José María Pego-Reigosa, Carlos Peña-Gil, David Rodríguez-Lorenzo, Irene Altabás-González, Naír Pérez-Gómez, John Henry Guzmán-Castro, Rodrigo Varela-Gestoso, Reyes Díaz-Lambarri, Alberto González-Carreró-López, Olga Míguez-Senra, Julia Bóveda-Fontán, Ángeles Charle-Crespo, Francisco Javier Caramés-Casal, Ceferino Barbazán-Álvarez, Íñigo Hernández-Rodríguez, Francisco Maceiras-Pan, Marina Rodríguez-López, Rafael Melero-González, José Benito Rodríguez-Fernández

**Affiliations:** 1 Rheumatology Department, University Hospital of Vigo, Vigo Health Area, Alto do Meixoeiro s/n, 36200 Vigo, Spain; 2 IRIDIS (Investigation in Rheumatology and Immune-Mediated Diseases) Group, Galicia Sur Health Research Institute, Alto do Meixoeiro s/n, 36200 Vigo, Spain; 3 Cardiology Department, Santiago de Compostela Health Area, Santiago de Compostela, Spain; 4 Service of Research, Education, and Innovation, Galician Health Service, Santiago de Compostela, Spain; 5 Quality Department, Vigo Health Area, Vigo, Spain; 6 Information Technology Department, Vigo Health Area, Vigo, Spain; 7 Primary Care Direction, Vigo Health Area, Vigo, Spain; 8 Health Information Department, Vigo Health Area, Vigo, Spain; 9 Pintor Colmeiro Primary Care Center, Vigo, Spain; 10 Primary Care Center of Porriño, Vigo Health Area, Vigo, Spain; 11 Primary Care Center of Marín, Marín, Spain; 12 Primary Care Center of Covelo, Vigo Health Area, Vigo, Spain

**Keywords:** Rheumatic diseases, Implementation, Early diagnosis, Telemedicine, Medical informatics, Needs assessment

## Abstract

**Objective:**

To describe in detail an innovative program based on telemedicine for semi-automated prioritization of referrals from Primary Care (PC) to Rheumatology, for reproducibility purposes, and to present the results of the implementation study.

**Methods:**

The context and situation were carefully analyzed, paying attention to all processes in place, referral numbers, waiting times, and number of complementary tests prior to discharge from Rheumatology. The composition of the team, aims, users, scope, and implementation phases were defined. Eight process indicators were established and measured before and 32 months after the program implementation.

**Results:**

The program, which includes IT circuits, algorithms based on response to specific guideline-based checklists, e-consultation, and appointments based on priority, was fully implemented in our health area after a pilot study in two PC centers. After implementation, 6185 rheumatology referrals showed an e-consultation response delay of 8.95 days, and to first face-to-face visit (after e-consultation) of 12.6 (previous delay before program implementation was 83.1 days). Resolution by e-consultation reached 20% (1195 patients did not need seeing the rheumatologist to have the problem solved), and 1369 patients (32%) were discharged after the first visit. The overall resolution rate was 44.0% (2564 discharges/5830 e-consultations). From a random sample of 100 visits, only 10% of patients needed additional complementary tests to make a diagnosis and decision by Rheumatology (20.9% decrease from previous period).

**Conclusion:**

A careful analysis of the situation and processes, with implementation of simple IT circuits, allows for the improvement of the efficiency and resolution of problems in Rheumatology.

**Supplementary Information:**

The online version contains supplementary material available at 10.1186/s12913-021-07455-4.

## Key messages

- Early and proper diagnosis is essential in inflammatory rheumatic diseases.
- Given the shortage of rheumatologists, this may require innovative healthcare models.
- A model based on IT circuits, checklists, e-consultation, and appointments based on priority, helped improve process indicators significantly.

## Introduction

Rheumatic and musculoskeletal diseases (RMDs) are highly prevalent; up to 28.9% of the population would consult their doctor for one such condition in any given year [1]. RMDs pose a significant challenge in terms of health resources, workplace absenteeism, and socio-economic impact. RMDs affect the lives of workers, both personally and through family members they must take care of [2].

Despite their frequency, RMDs are often not properly addressed, unless seen by rheumatologists [3]. However, studies show a trend towards a decrease in the number of specialists available [4–7]. This shortage of rheumatologists is counterintuitive, given the increase in demand for rheumatological care as population ages [8]. As a way to absorb the demand and to avoid overloading the health system, Primary Care Physicians (PCPs) provide rheumatological care and refer as few patients as possible to Rheumatology. Many times, this strategy results in delayed important diagnoses [9].

The 'traditional' model of referral from Primary Care (PC) to specialized care works on a first-come, first-served basis [10]. Referrals to rheumatologists range widely from routine referrals for soft-tissue conditions, where non-rheumatology care may be more appropriate, to urgent cases (e.g., systemic vasculitis or systemic lupus erythematosus flare). Inefficiencies in the scheduling and delivery of care through this referral model can result in a delayed access to first consultations. This may affect patients in need of priority assistance, either to palliate an acute disability, or to minimize chronic damage [7, 10–12]. As a paradigm, accurate and rapid recognition of patients with rheumatoid arthritis and other types of inflammatory rheumatic diseases is essential, as early diagnosis and intervention improve long-term outcomes [13]. Given the current scenario in relation to the shortage of rheumatologists, innovative healthcare models are required [14].

In the Vigo Health Area (North West of Spain), we have implemented one such innovative pathway to improve the referral process of patients with RMDs by applying information technology (IT) tools and telemedicine to improve communication between the PCPs and the Rheumatology team. The main aims of this telehealth program are the prioritization of referrals in a sensible way, the optimization of time to Rheumatology consultation, and the improvement of clinical problem resolution. In this article, we describe the program in detail and present the results of the implementation study so that it can be replicated and adapted elsewhere.

## Materials and methods

The core elements to support the implementation of an innovative health program are the context or environment, the analysis of the situation, the implementation team, the definition of aims, users and scope, program phases, and evaluation [15, 16].

### Context

The geographical location of the program is the Vigo Health Area in North West Spain, in which the central referral hospital is the University Hospital of Vigo. In 2012, the area population was 427,504 (206,686 men and 220,818 women), of whom 74,131 were under 15 years of age, 282,655 were in the15 to 64-year range, and 70,719 were 65 years old or older. Life expectancy of this population was 83.7 years [17]. Galicia’s economy - the Autonomous Community of which Vigo is the largest city - is based mainly on services (67.6%), followed by manufacturing (14.7%), agriculture (5.9%), and construction (5.6%). The unemployment rate in that year was 6.3% [17], which falls within the national average.

### Analysis of the situation

**Fig. 1:**
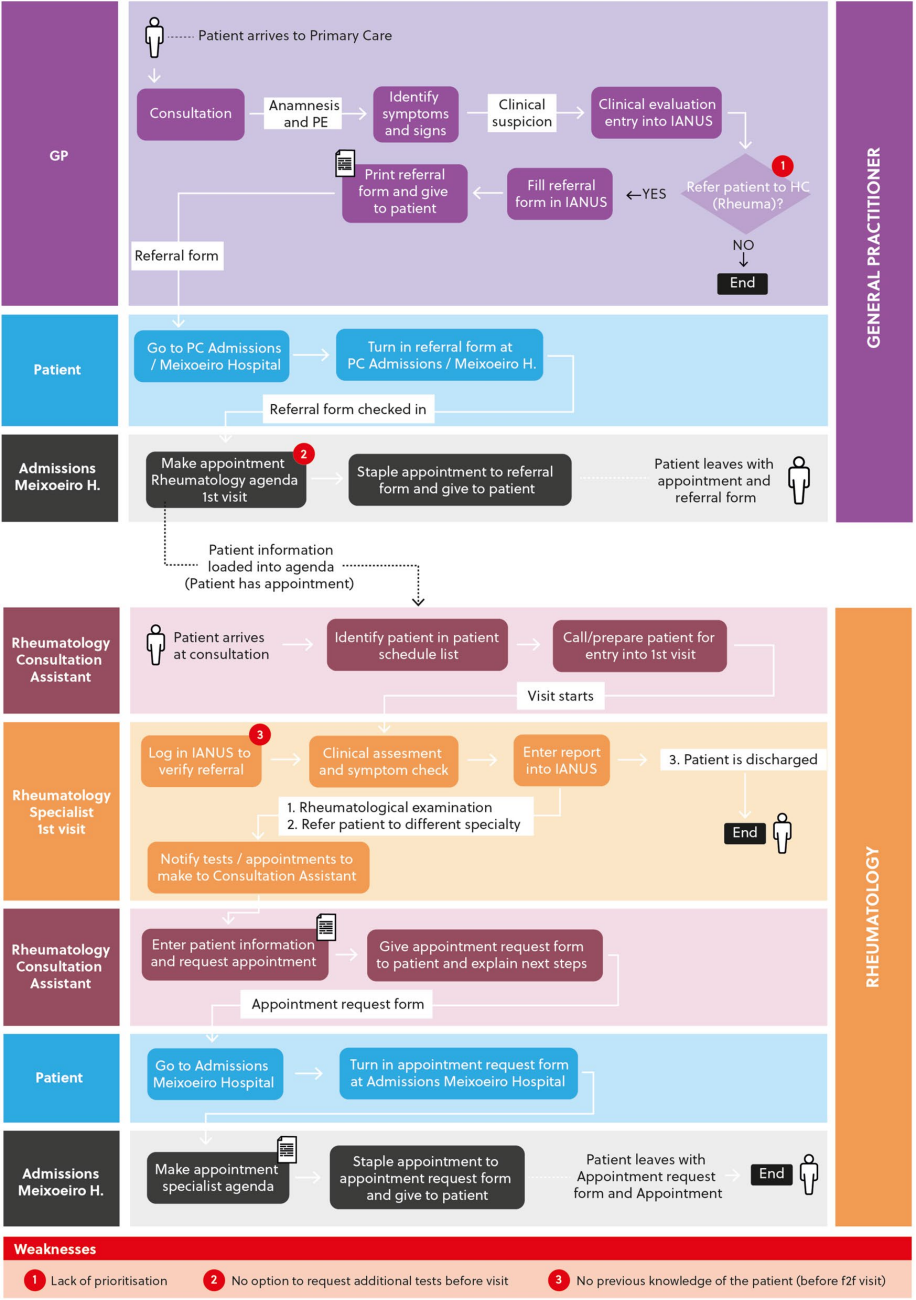
Process before the implementation of the innovative IT solution: original referral circuit and consultations from Primary Care to Rheumatology. PCP: Primary Care Physician; PE: physical exam; IANUS: electronic health record system in Galicia; PC: Primary care. The patient is referred to Rheumatology without a prioritization or without further request of tests. When seen (face-to-face) in Rheumatology, the patient is completely unknown to the rheumatologist

Prior to the implementation of the program in 2013, the healthcare organization model of the Vigo Health Area was based on specific services provided by different specialists who had hardly any previous communication or coordination with PC. The reasons for referral to Rheumatology varied enormously, from the need of a bone densitometry to be requested by the specialist to a suspicion of complicated lupus. Before the implementation of the program, no specific referral protocols were applied. The previous communication model between primary and specialized care and in between specialists, was a printed or electronic referral form that placed the patient on the waiting list without the specialists having had access to their clinical record beforehand. The full process, integrated in the electronic health record system in Galicia (IANUS), is depicted in Fig. 1.

Over a 9-month period (September 2012 to May 2013), before the implementation of the program, the number of referrals from PC to Rheumatology was 3115, an average of 388.2 patients/month. In the same period, the average waiting time for that first visit was 83.1 days, with a minimum value of 0 and a maximum value of 469 days. The percentage of patients who were discharged from Rheumatology after the first visit was 30%.

### Establishment of the team

The implementation team members were designated by the medical director and Rheumatology. The team was made up of rheumatologists and PCPs, along with computer and innovation experts (listed in the Appendix). A grant made it possible to contract a rheumatologist part-time to monitor and develop this innovative e-health program during a 12-month period.

### Program definition, aims, users, and scope

The aims, users and processes of the program were established on the basis of the analysis of the baseline situation, which was discussed in-depth by the team.

The aims of the program were: 1) to improve the screening of patients with RMDs to establish cases who may need priority care, and 2) to make communication between Rheumatology and PCPs more fluent.

The system was conceived to be used by PCPs and rheumatologists from the health area. All referrals - which were paper-based in the past - were turned into electronic referrals on the rheumatologists’ patient agendas. The transition from paper-based to electronic was carried out progressively and regular checks were performed before this was further advanced.

The target population of the program was that of the Vigo Health Area (427,505 in 2013) with suspected RMDs. Extrapolating from nationwide epidemiological estimates, this would be a total population of over 94,000 [2, 18, 19] . However, only those with incident inflammatory diseases, or with subacute disability due to RMDs - around 3500 people [20–22] - were expected to be referred to Rheumatology.

The program is based on clear processes incorporated in the IT systems, well defined prioritization criteria and times, indicators, and reports.

### Implementation phases

Each aspect of the program - circuits, referral criteria, diagnostic tests to be carried out before the assessment by the rheumatologist, critical indicators, and the evaluation (July to September 2013) - was designed by consensus following a thorough analysis of the situation, areas to be improved and potential solutions.

In order to achieve seamless communication between healthcare levels, regular meetings were held at each of the PC centers with representatives of PC, Rheumatology and medical directors, to present the program and to introduce any changes or improvements suggested by PC.

In the next 3 months, the healthcare integration circuit between PC and Rheumatology was designed on and implemented with IT tools. A 3-month pilot program was carried out in two PC centers, covering a population of 80,586 people (October to December 2013).

The pilot program allowed for the finetuning of the IT circuit. During this phase, all PCPs in the area were given proper training. This included the standardization of procedures, the establishment of dynamic agendas, and guidelines on how to proceed in the e-consultation with specialists.

In January 2014, the program was implemented in the entire health area and all PCPs were informed that no other referral systems would be accepted other than those cases demanding urgent attention. These should be referred directly to the emergency department.

### Evaluation

Once the program had been implemented in all the health centers of the area, data were analyzed including all referrals from PC to Rheumatology from October 2014 to June 2017.

The objectives and indicators established to measure and control the implementation of the program are presented in Table 1.

**Table 1:** Indicators and results of the program from October 2014 to June 2017

Objective^a^	Indicator	Result
To establish a tool for the integration of care between PC and Rheumatology	Ratio of referrals from PC channeled through the new electronic system	100% of 6185
To establish a process for managing Rheumatology e-consultations from PC	Implemented process for managing the e-consultations received	In full
To identify patients referred from PC who should be attended preferentially by Rheumatology (prioritization)	Waiting days for priority pathologies (recent onset arthritis, suspected connective disease, etc.)	8.95 days (Previously 83.1 days)
To improve the performance of essential diagnostic tests	% of patients assessed face-to-face with the essential diagnostic tests already available	89%^a^ (Previously 68%)^a^ ^a^From random samples 2 months
To reduce waiting time of PC referrals to Rheumatology	Waiting days for all referrals (face-to-face and e-consultation)	12.6 days (Previously 83.1 days)
Virtual resolution of e-consultations from PC to Rheumatology.	n (%) of e-consultations that are resolved virtually	1195 visits (20%)
To improve the resolution of the first face-to-face consultation	n (%) of first face-to-face consultations that are resolved in a single act	1369 (32.3%) of 4240 patients seen after e-consultation
To reduce Rheumatology waiting time of patients referred from other specialties	Waiting days of patients referred from other hospital specialties to specialized rheumatological care	19.2 days (Previously 83.1)

^a^ For first referrals (e-consultations) from PC to Rheumatology service (*n* = 6185)

### Ethics committee

This study did not require Ethics Committee approval.

## Results

**Fig. 2:**
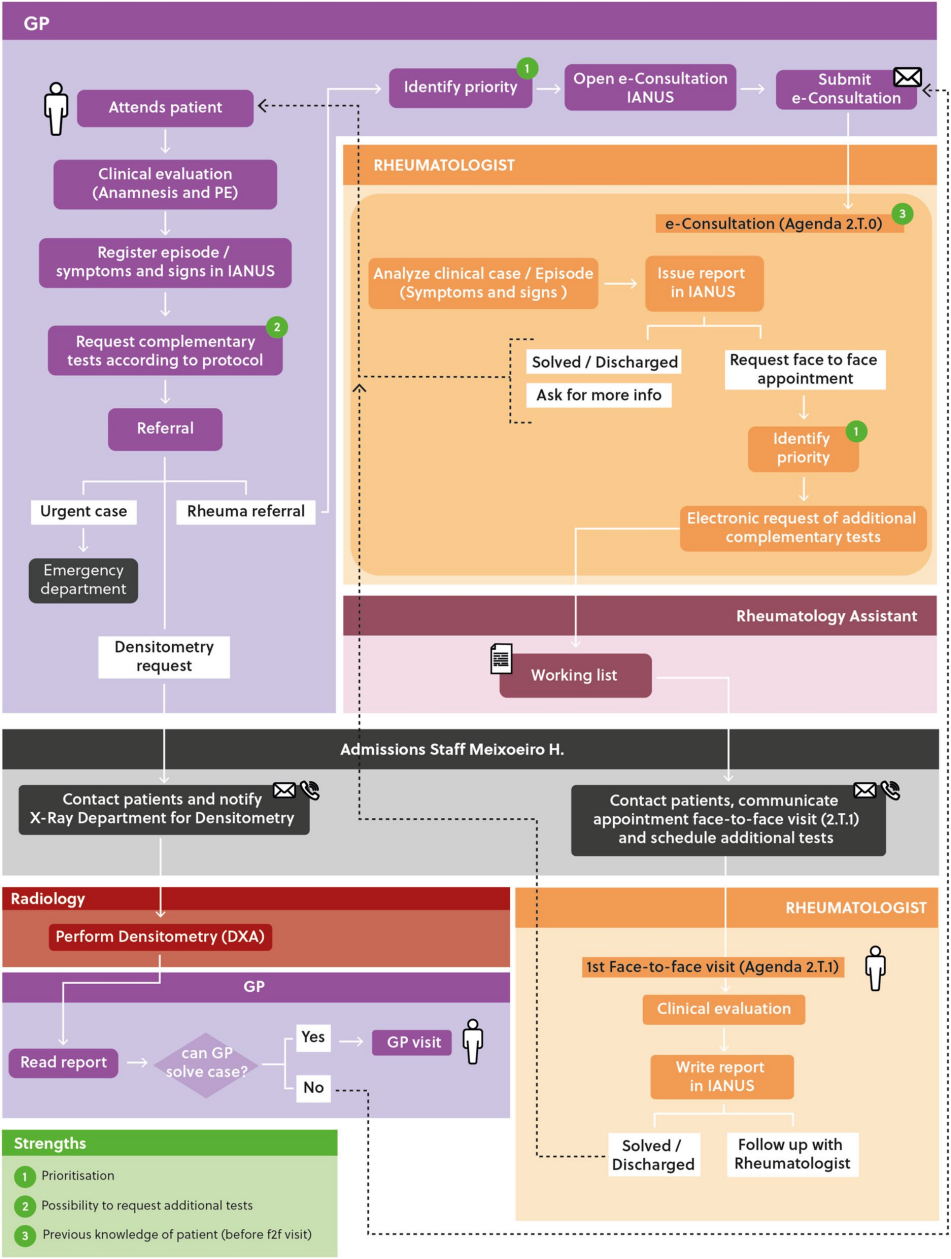
Process after the implementation of the innovative IT solution. The program: referral circuit, IT systems, and consultations in Rheumatology of patients referred from Primary Care. PCP: Primary Care Physician; PE: physical exam; IANUS: electronic health record system in Galicia. The patient is referred to Rheumatology, previous request of additional tests already agreed upon between PC and Rheumatology. The case is electronically seen by the Rheumatologist in the 2.T.0 Agenda, where he or she can add further tests or solve the case without a face-to-face visit. A second prioritization occurs here before the first face-to-face visit in Rheumatology (2.T.1 Agenda)

Figure 2 shows the clinical process circuit for rheumatology e-consultation. All processes and algorithms were agreed upon by PC medical directors.

**Fig. 3:**
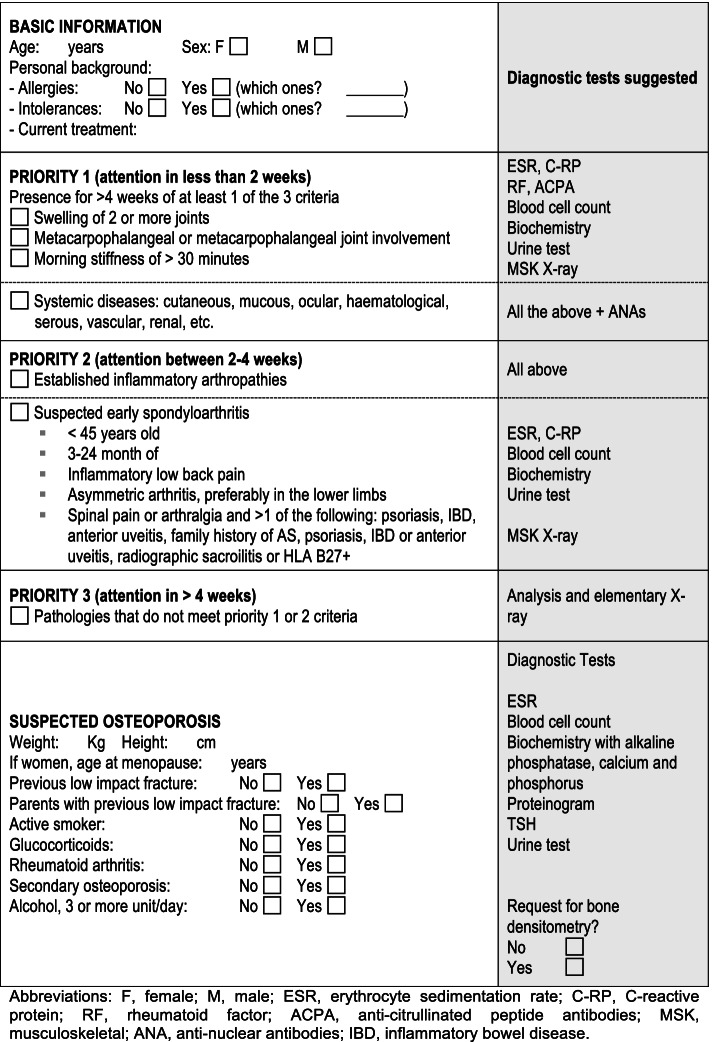
Referral screen (adapted for comprehension). F: female; M: male; ESR: erythrocyte sedimentation rate; CRP: C-reactive protein; RF: rheumatoid factor; ACPA: anti-citrullinated peptide antibodies; MSK: musculoskeletal; ANA: anti-nuclear antibodies; IBD: inflammatory bowel disease

In summary, any suspected RMD is recorded by the PCP onto the IT system (IANUS) and, following the pre-agreed criteria, priority of referral is established and the specific tests requested (Fig. 3).

The e-consultation request submitted by the PCP with a priority tag specifying the waiting time for an e-consultation (less or over 5 days) is received by Rheumatology. In the e-consultation, a rheumatologist analyzes the information provided together with the diagnostic tests and either advises the PCP on the way to proceed by means of an e-report, or sets a face-to-face appointment for the patient (sometimes, also requesting further tests electronically). Then, an assistant contacts patients to provide details about the date of appointment and possible further tests to be done before the face-to-face consultation. Some patients, i.e., those referred to bone densitometry are managed directly through the IT system via a specific circuit including a fracture risk score calculation inserted in the intranet. These patients are not seen face-to-face in Rheumatology unless the results of the densitometry are unfavorable, and osteoporosis is severe or secondary, in which case, an appointment is set by an assistant.

All PCPs of the area (100%) channeled referrals to Rheumatology through the proposed program (Electronic History and Electronic Request Manager).

### Post-implementation evaluation

In the 32 month-period of evaluation, a total of 6185 referrals (e-consultations) were sent from PC to Rheumatology. Table 1 shows the results of the evaluation.

The delay in response was 8.95 days, which means a reduction in the delay of the first consultation of 74.1 days (89.2%) with respect to the period analyzed prior to implementation in 2012. The delay until the first face-to-face visit was 12.6 days (a reduction of 70.5 days, or 84.8%).

The average percentage of e-consultations resolution reached 20.5% (a total of 1195 patients did not need to go to the hospital to solve their health problem; most of them were patients with suspected osteoporosis or who were referred to bone densitometry), while the average number of patients who did not need a second consultation after a first face-to-face visit was 32.3% (1369 patients). The overall resolution capacity between the two consultations was 44.0% (2564 patients not requiring an additional consultation /5830 consultations).

In relation to the requests for diagnostic tests (laboratory, X-ray and bone densitometry), the number of first visits to Rheumatology for which tests had not been carried out was analyzed in two randomly selected months, one prior to the implementation of the e-consultation process (April 2013) and the other after its implementation (April 2016). They totaled 608 in 2013 and 481 in 2016 (20.8 % reduction). After implementation, almost 90% of the patients had complementary tests performed from PC before their face-to-face specialist assessment. As a result, the request for complementary tests by the Rheumatology service before and after the implementation of the program decreased by 20.9% (608 vs. 481, respectively).

## Discussion

Before the program, patients referred from PC to Rheumatology had to wait for a long time before their first face-to-face visit. In the case of minor clinical problems, these could have been solved immediately, without requiring a visit to the hospital. After the implementation of this e-consultation program, waiting times were significantly reduced, while assuring prioritization of inflammatory diseases, and improving communication between health care levels. Furthermore, the overall optimization of the waiting list had a positive effect on referrals from other specialties as well as reductions in waiting times due to wider availability of consultation slots.

In general, most studies of health services in Rheumatology show a significant consultation delay of patients with inflammatory diseases [23–26]. Even though referral criteria and early management constitute a key aspect of the primary care and specialist interface [13], there are delays due to systemic inefficiencies in the referral process, scheduling, and care delivery, that may preclude access to specialist care, thereby increasing waiting times [10, 12, 14].

The situation in our area prior to the implementation of the integrated e-consultation program, was no outsider to such inefficient context. This could be defined by the following characteristics: 1) all referrals to Rheumatology were made for face-to-face visits; 2) absence of a prioritization scheme (i.e., a patient with a recent onset arthritis had to wait as much as a patient with suspected osteoporosis); and 3) many first visits to Rheumatology required additional consultation. Many of the patients referred to Rheumatology could have easily avoided the visit, many others requiring specialist attention had to wait too long for a visit.

The success of the implementation of the program was based on two fundamental aspects: the involvement and consensus of all stakeholders and the establishment of the electronic referral as the only way to access Rheumatology. All the information on the e-forms, including referral criteria and tests to be performed, as well as the circuits to be followed, had been agreed upon by the rheumatologists and PCPs in the area. The program was presented at each PC center by a representative of both the board of directors and Rheumatology. Also, the involvement of the IT Department since the beginning of the project made its implementation in the intranet accessible to all [27]. Patients could only be referred to Rheumatology through e-consultation, and the IT system included tools to help prioritize and diagnose in advance, which eased the way to both the PCPs and the rheumatologists. In summary, such a thorough process led to a successful implementation as previously suggested by other authors [28]. It is important to highlight at this point that there was an agreement not to apply this referral system to rheumatologic emergencies, such as suspected septic arthritis, or giant cell arteritis. In these cases, patients have to be referred by the PCP directly to the Emergency Department, which has to contact Rheumatology by telephone, or arrange an e-consultation within 24 hours.

A significant proportion of the attention to chronic diseases (among them, RMDs), would not require patients’ attendance by medical specialties and could be managed by PC if there was a channel of agile and immediate communication between this and the specialists. Even before the COVID-19 pandemic, it was obvious that many situations could be managed by Rheumatology without a face-to-face visit [29]. This has been made even clearer during the pandemic [30–34]. Nevertheless, we tend to think of telemedicine as a virtual visit, but there are other ways to solve a case referred by a PCP such as a chat, an e-mail or report, that do not involve the patient.

This type of consultation has a positive impact on both levels of care since mutual interaction, trust and learning between PCPs and rheumatologists are greatly enhanced.

It is necessary to prioritize patients’ attention based on adequate and reliable clinical information provided by the PCP to offer individualized care according to the particular situation and demand of each patient. This prioritization can be established by rheumatologists in a triage stage, through protocols previously agreed upon with the PCPs [10, 14, 24, 35, 36]. Better results can be obtained, saving the time used by the rheumatologist in consultations, by doing this triage with IT systems in place, as in our program.

Such a strategy should always be implemented in close coordination with the PCPs, this being the reason why they have been involved in every stage of the process.

One of the most outstanding aspects of our study is the reduction in waiting times for a first face-to-face visit. Before the implementation of our strategy, the average waiting time was 83.1 days, with virtually all patients within 2-3 months. Only very occasional cases had waiting times longer than one year (exceptional patients who were referred for review in 1.5-2 years and who were registered as first visits). Once our project was launched, the average waiting time for a first face-to-face visit was reduced by approximately 85%. A key aspect of any waiting time management strategy is the coordination between administration and triage of referrals. Much work has been done to define the best referral system. Single-entry models have been used successfully in some service industries, where users 'gather' at a single-entry point to access the first service available. These could represent a useful approach to reducing waiting times for health services when, for example, waiting times between consultants are variable, as patients who wait longer can be scheduled for the next available slot [37, 38]. Although there are benefits reported for centralized referral systems, these apply to homogenous patient populations requiring a specific procedure. However, their positive impact on the population of RMD patients, characterized by a high variability in diagnosis and urgency of referrals, is not clear [14]. This is where our work offers a real innovation. By evaluating all the reasons for consultation, on the one hand we give greater autonomy to PCPs (who can now order directly a bone densitometry, for example) and, on the other hand, we give priority to patients with more serious conditions. All this takes place in a collaborative environment between PCPs and specialists for the resolution of cases with bidirectional e-consultations. Rheumatology and PC professionals, despite the fact that they had to learn how to use a new IT tool, soon discovered that it was also useful for rapid patient assessment. For the PCP, screening tools are always helpful and become true learning experiences. At the organizational level, the institution had already a common integrated electronic medical record between PC and Rheumatology, but there was clear room for improvement, especially at referral level.

Other aspects of the evaluation of implementation are still missing. Although our perception was that both provider and patient satisfaction were really high, we have not assessed the experience of patients and providers with this process, or safety outcomes. Unfortunately, as we had no prior indicators for these aspects before the program implementation, a comparison was not possible. On the other hand, we did not have excessive problems from the PCPs when implementing our project, likely due to the previous explanation of its characteristics and the degree of consensus reached. The greatest reluctance came from the rheumatologists, in relation to the change in working procedures. This initial resistance was overcome when they began to perceive the advantages that the strategy entailed both for the patient and for the services involved in it.

From a cost-effectiveness point of view, and after the 12-month part-time period allocated by a rheumatologist to its development and implementation, the program has not involved any additional financial costs to the Public Health System. Even when we did not perform a formal economic evaluation, using the technical resources already existing in the system, not requiring the hiring of personnel and avoiding the displacement of the patient, is inescapably associated with saving money.

Despite its encouraging results, our work may have some limitations. The fact that it has focused only on the Vigo Health Area may limit the extrapolation of the results. In turn, our program could be considered for implementation by other health areas. Another possible limitation is the certain degree of digitalization required, which in some places can be a problem.

In summary, innovative circuits and e-consultation tools widely agreed upon by users achieved a more efficient referral from PC to Rheumatology in the Vigo Health Area. Patients requiring priority consultation are now assessed in the shortest time possible. All other patients wait only a little longer in favor of the first. In addition, the communication between PCPs and rheumatologists was strengthened and facilitated, providing the former with greater resolution capacity.

## Supplementary Information

**Additional file 1.**

## Data Availability

Data sharing is not applicable to this article as no datasets were generated or analysed during the current study.
