# Supplementary material for: Analysis of the implementation of an innovative IT solution to improve waiting times, communication with primary care and efficiency in Rheumatology
Source: BMC Health Serv Res. 2022 Jan 12;22:60. doi: 10.1186/s12913-021-07455-4 (PMC8754366; doi:10.1186/s12913-021-07455-4)
Supplement: Supplementary file 1 — Additional file 1. [file 12913_2021_7455_MOESM1_ESM.docx]

**Appendix: Implementation team.**

The members were all from the Vigo Health Area, unless otherwise indicated:

- Process Engineer: Carlos Peña Gil (Santiago Health Area)
- Quality and Innovation Technician: David Rodríguez Lorenzo
- Information Systems:
  - Rodrigo Varela Gestoso (Vice-Director of the Information Technology Department)
  - Alberto González-Carreró López (Head of Health Information Systems Unit)
- Administration: Olga Míguez Senra (Section Chief)
- Medical Directors:
  - José Benito Rodríguez Fernández
  - Francisco Javier Caramés Casal
- Primary Care Physicians
  - Julia Bóveda Fontán
  - Ángeles Charle Crespo
- Rheumatologists
  - Ceferino Barbazán Álvarez (Coordinator of Rheumatology Department) Íñigo Hernández Rodríguez (Section Chief)
  - Francisco Maceiras Pan
  - Marina Rodríguez López
  - Rafael Melero González
